# Providing family planning services to women in Africa

**DOI:** 10.2471/BLT.17.020917

**Published:** 2017-09-01

## Abstract

Access to contraceptives in Africa has not increased at the same pace as elsewhere in the world. Some African nations are investing in family planning services to reduce fertility rates, improve economic development and their population’s health. Tatum Anderson reports.

Berhane Assefa, national family-planning coordinator in Africa’s second most populous country, Ethiopia, recalls that only 20 years ago many women in her country gave birth to more than eight babies.

By 2016, however, the average fertility rate had fallen to 4.6 live births per woman, according to Ethiopian Demographic Health Survey (DHS), and in Addis Ababa, the capital of this country of some 99 million people, women have fewer than two babies on average, Berhane says, citing the Ethiopian DHS 2011.

“Every five years the contraceptive prevalence (proportion of women using contraceptives) is doubling, so I am happy with the programme because more women can manage the size of their families,” she says.

“I am happy with the programme because more women can manage the size of their families.”

Berhane Assefa

Ethiopia’s family-planning revolution started about 10 years ago when hundreds of primary care centres were built, contraceptive stocks increased and the health extension workers based in these centres were trained to advise families on contraceptive methods.

These community health workers started to distribute male and female condoms, provide medroxyprogesterone acetate injections and, more recently, they have been trained to insert and remove implants and intrauterine devices.

Awareness-raising and education have been key to helping women and their husbands decide how large their families should be, when to stop having children, and the options available to achieve this.

The results are impressive. Between 2000 and 2016, the proportion of married women using contraceptives increased from 8% to 36%, according to the Ethiopian DHS 2016.

Ethiopia has become part of a family-planning revolution that has seen the use of contraceptives taking hold across the globe since the 1960s.

Family planning the world over has long been fraught with political, cultural and religious controversy, ever since the first pioneers began to promote the benefits of birth spacing in the early 20th century.

In spite of these controversies, huge progress has been made.

Between 1950 and 1975, average global fertility rates remained steady at five live births per woman. But as contraceptives and sex education became increasingly available – along with economic development, more women in the workplace, changes in social mores and increasing child survival – that figure halved to an average of 2.5 births by 2015, according to United Nations data.

The unmet need for contraceptives among married women aged 15–49 years in WHO’s Africa Region is estimated at 24% and lags considerably behind the rest of the world, according to the *Atlas of African health statistics 2016.*

Religious opposition to family planning has been at the heart of many restrictions, notes Dr Nafis Sadik, executive director of the United Nations Population Fund (UNFPA) from 1987 to 2000.

In the 1960s, Sadik implemented one of the world’s first national family-planning programmes in a developing country, in Pakistan. As Secretary-General of the landmark 1994 Cairo International Conference on Population and Development, she guided the conference preparations and proceedings.

She recalls meeting the late Pope John Paul II to discuss the Vatican’s opposition to all contraception a few months before the conference in 1994. “Somehow, I thought I could change his view,” she says.

“The only thing that he accepted to prevent pregnancy was abstinence, but that was not always possible for women … and there was immense social pressure on most marriages to have children.”

The debate became so divisive that even the UN Millennium Development Goals initially omitted family planning in 2000, although it was included, as an indicator to monitor progress in 2007, when the benefits for development and women’s rights were recognised.

Today, the efforts of public health advocates like Sadik have paid off and family planning is no longer widely seen as a tool of population control, but rather an essential component of development and a means to promote and protect basic human rights.

“Family planning started as a population control idea, but then it changed,” says Sadik, adding that the 1994 conference helped to establish the idea that family planning addresses primarily concerns about health and rights.

For Ian Askew, Director of the Department of Reproductive Health and Research at WHO, the most dramatic transformation in family planning over the last 30 years has been the rapid change in the norms around family size and attitudes to contraceptive methods.

“Most people don’t want four or more children any more, today they want one or two, or sometimes three children,” he says.

“People realise that smaller families are generally healthier, more prosperous and have better educated children and – in most countries – people no longer feel social or cultural pressures to have so many, because women are less likely to die in childbirth and child survival is much greater,” he says.

Education, especially of women and girls, is key. High literacy rates in Zimbabwe, for example, mean that family planning is widely appreciated and used.

With one of the highest contraceptive prevalences in sub-Saharan Africa – 67% of married women aged 15 to 49 years, according to the 2015 Zimbabwe DHS – the country exceeds the global average, according to Dr Munyaradzi Murwira, Executive Director of Zimbabwe National Family Planning Council.

“Today women are able to get an education and have a career. This results in smaller families in Zimbabwe,” he says.

In the United Republic of Tanzania, women who have just given birth are offered counselling and contraceptives while they are still in hospital to promote child spacing, says Halima Shariff, Director of Advance Family Planning.

She adds that the Ministry of Health and its partners have played a key role in running campaigns to educate the public about family planning and its many benefits.

“Outreach programmes to deliver services have done a great deal in terms of changing the landscape and communicating the benefits of family planning,” Shariff says.

“Religious and cultural practices may militate against the smooth provision of these services, but this resistance is gradually subsiding.”

Halima Shariff

“As in many countries in sub-Sahara Africa, religious and cultural practices may militate against the smooth provision of these services, but this resistance is gradually subsiding,” Shariff says, adding that her country is striving to attain a national contraceptive prevalence target of 45% by 2020. Prevalence currently stands at about 32%, according to the country’s DHS 2015–2016.

These commitments announced at the Family Planning Summit in July in London, underscore the Tanzanian government's and its partners’ determination to prioritize family planning in the country's development planning process, she says.

For the advocates and implementers of family planning, it remains unclear how a recent policy change by the United States government to stop funding family planning services that provide safe abortion services will affect programmes across Africa.

To plug the resulting funding gaps, an initiative called “She Decides”, launched by the Netherlands government, sprung up to reinforce women’s rights to decide freely whether to have children, when and how many to have.

Potential funding gaps are nothing new, as funding for family planning was diverted to HIV programmes across the developing world during the 1990s.

“Funding for our family planning programme has been limited over the last 10 to 15 years,” said Murwira. “That is the biggest challenge we have faced.”

Despite religious, cultural and political opposition to family planning, contraceptive use has increased across Latin America, where Catholicism predominates, in the largely Muslim nations of the Eastern Mediterranean, and in Asia and southern Africa, showing that the desire to plan pregnancies is often stronger than belief systems.

Barriers to meeting the full demand for contraceptives abound. Stockouts limit access to contraception; some countries limit contraceptives on non-medical grounds, for example, to unmarried women and adolescents; and some women need their husbands’ consent to use contraception or must pay for it.

For James Kiarie, Coordinator of the human reproduction unit at WHO headquarters, the high unmet need is not surprising. Most governments could invest more in family planning services or use mass media more effectively to challenge myths claiming that contraceptives make wives more promiscuous, cause cancer or are against the will of God.

“Such myths can circulate if unchallenged,” Kiarie says.

The Family Planning 2020 partnership, launched in 2012 by the United Kingdom of Great Britain and Northern Ireland, UNFPA, the Bill & Melinda Gates Foundation and many others, aims to increase investment in the 69 poorest countries so that 120 million additional women can meet their contraceptive needs by 2020.

**Figure:**
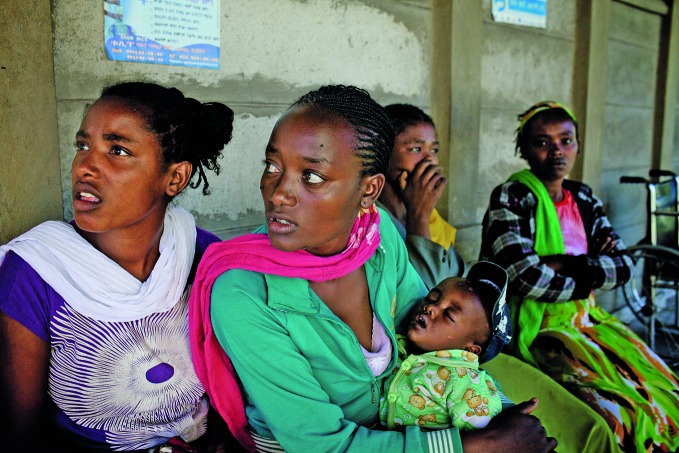
Women wait for family-planning services at a health centre supported by the International Medical Corps in the Damot District in Ethiopia. The programme is funded by the European Commission.

© European Commission/Ashley Gilbertson

**Figure:**
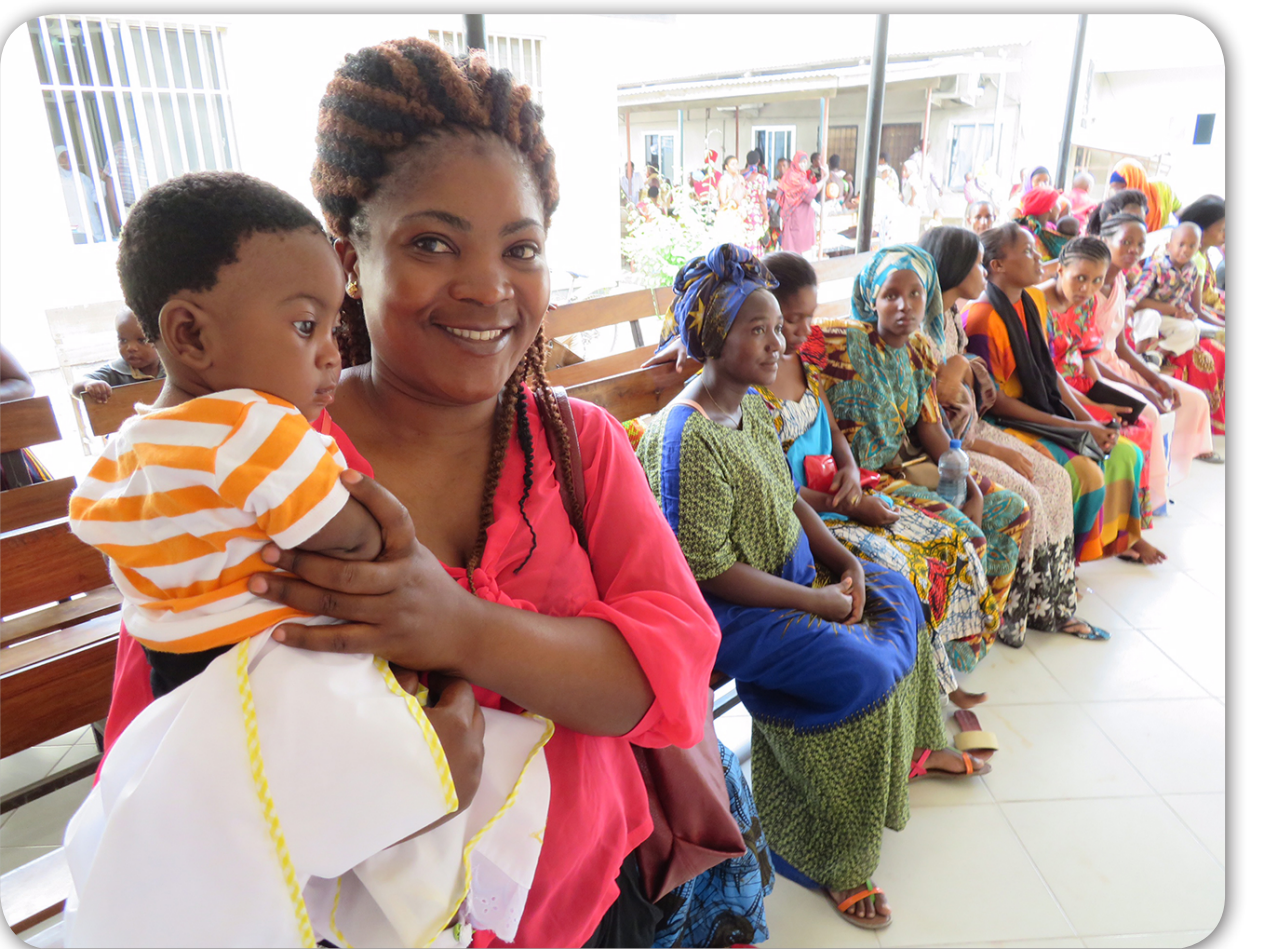
Women attending a local family planning services clinic in Dar es Salaam. The clinic is funded by the UK Department for International Development (DFID)

DFID/ Jane Miller

